# Validation of the Tunisian Social Situation Instrument in the General Pediatric Population

**DOI:** 10.3389/fpsyg.2020.557173

**Published:** 2020-10-29

**Authors:** Olfa Rajhi, Soumeyya Halayem, Malek Ghazzai, Amal Taamallah, Mohamed Moussa, Zeineb Salma Abbes, Malek Hajri, Houda Ben Yahia, Maissa Touati, Radhouane Fakhfakh, Asma Bouden

**Affiliations:** ^1^Department of Child and Adolescent Psychiatry, Razi Hospital, Manouba, Tunisia; ^2^Faculty of Medicine of Tunis, University of Tunis El Manar, Tunis, Tunisia; ^3^Department of Preventive Medicine, Abderrahmen Mami Hospital, Ariana, Tunis, Tunisia

**Keywords:** theory of mind, social cognition, validation, autism spectrum disorder, factor analysis

## Abstract

**Background:**

In order to better understand the deployment of the theory of mind (ToM) in Tunisian neurotypical children, we have developed a new tool of assessment of the ToM called the “Tunisian Social Situations Instrument” (TSSI). We opted for the creation of this test in view of the intercultural differences in the development of social skills. Our purpose was to validate this tool in general pediatric population.

**Methods:**

It was a cross-sectional evaluative study that aimed to validate the TSSI in the general pediatric population. We initially conducted a beta test and a pre-validation study before taking the initial version of the TSSI on 123 neurotypical children. Then, we followed the typical validation procedure: appearance validity, content validity, construct validity, and reliability study.

**Results:**

Regarding the validity of appearance, the TSSI was comprehensible and adapted to the Tunisian pediatric population. About content validity, the exploratory factor analysis extracted 6 factors that explain 69.3% of the total variance. These factors were respectively social clumsiness types 1 and 2, intention attribution, emotional ToM, epistemic ToM, and simple comprehension questions. The subdomains of social clumsiness (types 1 and 2) and emotional ToM had a Cronbach alpha higher than 0.8. This factor structure as well as the significant inter-correlation between subdomains and the global score were in favor of a good construct validity. The internal consistency study showed good reliability of the final version of the TSSI (alpha of Cronbach at 0,809). Regarding the performance of children at the TSSI, we have noticed a significant association between the global score, age, and verbal intelligence.

**Conclusion:**

This work offers valuable insights about ToM and provides clinicians with a reliable tool to assess social clumsiness and emotional ToM in typically developing children.

## Introduction

The theory of mind (ToM) is a crucial domain of social cognition. It is essential for meaningful human social interactions. It consists of the ability to attribute mental representations to oneself and to others and to thereby interpret and predict their behavior (Benson and Haith, 2010).

The concept of ToM was first proposed by the primatologists Premack and Woodruff (1978). Based on their study on non-humans, they attempted to identify an individual’s ability to assign mental states to himself and to others (thoughts, desires, intentions, emotions). The term “theory” was used to explain the fact that mental states are not observable and that their understanding involves a system of inferences that can be used to make predictions about others’ reactions (Premack and Woodruff, 1978).

Theory of mind has recently attracted attention within the field of social neurosciences, which has generated a large body of consensual literature regarding the processes underlying this social competence (see for review Beaudoin et al., 2020).

Scholl and Leslie (1999) defended the modularity, also called the specific mechanism theory. This approach states that an innate ToM module is working by the second year of life. The development of the theory of mind depends on the neurological maturation of certain brain structures (Mahy et al., 2014).

To examine the mechanisms behind ToM and their change over time in a more fine-grained manner, researchers have turned to neuroimaging of ToM related tasks. This research, by Frith and Frith (2003) had not only descriptive aims but also theoretical purposes: it supported the modular model of ToM (Gallagher and Frith, 2003).

In addition to the different theoretical models of ToM, researchers have been also interested in describing meaningful distinctions between ToM types. Whereas “hot” ToM requires an understanding of others’ emotions, affective states, or feelings, “cold” ToM requires an understanding of their cognitive states, beliefs, thoughts, or intentions (Brothers and Ring, 1992). These two concepts have also been differentiated in terms of their neurofunctional bases as well as in terms of cognitive tests (Schurz et al., 2014).

To elucidate the cognitive also called epistemic ToM, Coricelli (2005) introduced two levels of understanding of others’ intentions and behaviors. The first level refers to automatic preconceptual phenomena that specify a primitive understanding of another person’s mind. The second level of mindreading is conceptual and voluntary. Its acquisition involves the ability to adopt the perspective of the other person in the understanding and predicting of behavior (Coricelli, 2005).

Furthermore, the ability to reason about second- and higher-order beliefs and to understand multiple perspectives within a communicative situation involves advanced ToM. It is a more mature ToM ability that differs from simple false belief understanding or detecting an affective mental state. It is needed in complex and ambiguous social situations, especially when they require differentiation between cognitive and affective mental states (Białecka-Pikul et al., 2017).

Several tools for evaluation of the theory of mind have been designed and validated. False belief tasks investigate first- or second-order ToM. They assess the ability to understand a person’s beliefs about a state of the world, whereas the latter is the ability to infer nested mental states and to understand a person’s beliefs about someone else’s beliefs (Bosco et al., 2016). Happé’s (1994) (White et al., 2009) and “Reading the Mind in the Eyes’ Task” are classic validated tasks that assess advanced ToM (Jolliffe and Baron-Cohen, 1999). Baron-Cohen (1999) proposed the so-called Faux Pas test (Baron-Cohen et al., 1999). It assesses the ability to detect a declaration or an action that unintentionally offends others. This test has been adapted and translated into French, Portuguese, and Swedish (Söderstrand and Almkvist, 2012; Garrigues, 2013; Faísca et al., 2016).

In our work, we relied on the modular approach of ToM and the preestablished tools of its assessment to design our test entitled TSSI. The theoretical subdomains of ToM treated by the TSSI were cognitive ToM (attribution of intentions, false belief detection), affective ToM, and advanced ToM (detection of Faux Pas). We also added simple comprehension questions and control situations.

The universality of intentional reasoning and the attribution of epistemic and affective mental states does not, however, resolve the question of cultural variability (Williams et al., 2003; Mc Grath, 2009; Wang et al., 2016). ToM, also referred to as “commonsense psychology” or “folk psychology,” depends closely on historical, socioeconomic, and cultural factors. This social competence is cultivated in a context of social interaction, displaying culturally specific developmental routes.

Contrasts in the developmental trajectory of ToM (Wang et al., 2016) have been reported between children from different parts of Europe: the United Kingdom and Italy. In the first cross-cultural study of theory of mind (Hughes et al., 2014), compared means on a battery of theory-of-mind tests in school-aged children from the United Kingdom, Italy, and Japan matched on age, gender, and verbal ability. Key findings were that there was U.K.–Italy contrast and a delay in Japanese children. These differences were explained by the importance of the pedagogical experiences and the cultural specific epistemologies that shape mental state inferences.

It is therefore essential that one keeps these factors in mind during assessments, both in selection of appropriate tests and in interpretation of performance. All cognitive and social tests not only need to be translated but also must be culturally appropriate, and local normative data should be used over the Western norms provided with the tests (Hamilton et al., 2016).

With the lack of equivalent tool of ToM, and through an intercultural perspective, we opted for developing a new test of ToM adapted to the pediatric Tunisian population. In this paper, we present the internal validation of the new ToM evaluation tool entitled “Tunisian Social Situations Instrument”’ in the general pediatric population. It is a comprehension test that evaluates the attribution of epistemic and emotional intentions and mental states to the protagonists of the social situations.

Thus, the aims of our research were to validate the TSSI in the general population among Tunisian children aged 7 to 12 with two main hypotheses: test scores improve with age, and test results are correlated with verbal intelligence. In fact, the quality of language is a relevant factor when considering the development of the ToM. Through a social constructivist perspective, participation in linguistically mediated conversations or narratives contributes to children’s discovery of the mind. Studies of typically developing children have also supported the general language hypothesis (Farrar and Maag, 2002). Therefore, it is recommended that ToM assessment is preceded by language evaluation and that its score is correlated with verbal age.

## Materials and Methods

### Participants

We conducted an evaluative cross-sectional study in the general pediatric population. We included children enrolled in ordinary schools, speaking Arabic and more precisely the Tunisian dialect, aged between 7 and 12 years. We did not include children with school failure, or with a present or past psychiatric disorder diagnosed by the examiners when administering the MINI K-SADS-PL for School-Aged Children-Present and Lifetime Version.

Children with an ASD, intellectual disability, attention-deficit/hyperactivity disorder sensory, or neurological deficits were also excluded from this study because these disorders interfere with the test performance (Hamilton et al., 2016; Hutchins et al., 2016).

We carried out an exhaustive study in five school child daycare centers, four primary schools, and a cultural center in five Tunisian states (Kef, Nabeul, Tunis, Ariana, Manouba).

During the β study, we administered the first version of the digital test to 15 participants. This step enabled us to send developers some suggestions and comments about the application. Then, we conducted a pilot study that included 20 typically developing children.

Of the 150 recruited children for the validation study, five children who were diagnosed with specific learning disorders and post-traumatic stress disorder were not included. Twenty-two children were eliminated by the pretest (results of the categorical analysis and/or of B vocabulary of the EDEI-A in its version adapted to the Tunisian population (Ben Rejeb, 2003) lower than expected for chronologic age). Thus, the final number of the participants included in the validation analysis was 123 children.

The socio-demographic characteristics of these samples are summarized in Table 1.

**TABLE 1:** Socio-demographic characteristics of β, pilot, and validation studies.

**Study**	**Sample size**	**Mean age**	**Sex ratio**	**Age distribution (years)**					
				**7**	**8**	**9**	**10**	**11**	**12**
β Study	15	8.5 years	0.36	2	3	4	2	2	1
Pilot study	20	9 years ± 6 months	1.5	3	5	3	6	3	0
Validation study	123	9.6 years ± 1.4 months	0.8	10	18	33	19	31	12

### Material

The TSSI is a test of comprehension composed of 10 social situations evaluating the attribution of intentions and epistemic and affective mental states to the protagonists of the social stories. It has been designed as a downloadable application on android (on tablet or personal computer or mobile phone). We opted for a digital design of the test because it made it more convenient and more attractive for children. It is also time-saving since it reduces time required to pass the test and to input and save data. Also, we have opted for this digital form to ensure its reproducibility and to make it easier to modify its content (by correcting the algorithm by the developer of the application). Within the limits of taking the test in its paper–pencil version during the β study, we did not observe any difference in the speed or in the nature of the answers compared to the digital form.

Each situation includes a text written in Tunisian Arabic dialect illustrated by one or more pictures with a synchronized reading of the text and questions. The reading of the texts and the questions is done automatically. The examiner inputs the child’s answers on the android device.

The social situations were inspired from The Faux Pas of Baron-Cohen et al. (1999) and Baron-Cohen et al. (1999), Sally and Anne test in its original (Girli and Tekin, 2010), and revised version suggested by Riviere in a personal communication (Baron-Cohen et al., 1999) and the strange stories of Happé (1994).

The initial version of the TSSI (see Appendix 1 and Appendix 2) consisted of 10 situations or tasks numbered from 1 to 10, including three control situations (stories 1, 8, and 10) and 7 containing a Faux Pas or a false belief. We included control stories in order to better assess the child’s skills to discriminate between both kinds of situations. The protagonists of the stories are 3 children (Salma, Rami, and Myriam) and an adult (Salma’s mother). The general theme of the 10 stories is about Salma’s birthday party (see Appendix 1). A demonstration entitled Situation 0 is used to prepare the child for the test and to explain the notion of Faux Pas and false beliefs. The answers to situation 0 were not included in the overall test score. One to four questions are asked in each situation to evaluate the child’s comprehension and the detection of the Faux Pas and/or false belief and the attribution of intentions and epistemic and affective mental states. Each answer was rated 1 if it was correct and 0 if it was wrong. We obtained a score for each situation and an overall score for the whole test out of 25 (see Appendix 2).

### Procedure

The study protocol was previously approved by Razi Hospital Ethics. Then, we got the authorization of the headmasters of the primary schools and the director of the cultural center in which the test was taken. Third, the parents of the participants were required to read and sign an informed consent form explaining the aim of the study and the confidentiality of the data. Fourth, participants underwent the pretest consisting in categorical analysis and vocabulary B tests of EDEI-A (Ben Rejeb, 2003). Socio demographic data and the respective pretest results were input into the “Form” application. Finally, the TSSI was administered individually in an isolated and quiet room. The overall procedure lasted about 30–45 min.

### Statistical Analysis

Statistical data were input and analyzed using the Statistical Package for Social Sciences (SPSS) program in its 23^*rd*^ version for Windows. Quantitative variables were described using means, standard deviations, and limits. Qualitative variables were described using proportions and percentages. We performed a MANOVA to study the difference in mean scores by age group and used Student’s *t*-test to study test scores according to the gender and age of the children. To study the internal structure of the TSSI, we performed an EFA with Varimax rotation. Correlations between item scores and the overall score as well as the inter-correlation matrix were calculated using Pearson’s r-correlation coefficient. We used Cronbach’s alpha index (Vaske et al., 2017) to study the internal consistency of the TSSI. The significance threshold chosen was “*p* < 0.05.” We also performed a multiple-regression analysis to rule out the role of the age in ToM performance.

## Results

### Validity of Appearance

#### Expert Opinion

A group of four child and adolescent psychiatrists, three clinical psychologists from Razi Child and Adolescent Psychiatry Department (Tunisia), and the developer of the TSSI application judged our tool and suggested some adjustments for its exploratory version.

Regarding the visual support (pictures illustrating each situation), graphic details were proposed to offer non-verbal clues for a better understanding of each situation, such as facial mimics of the characters, their gestures, and other graphic details related to the context of each situation.

The wording of the texts and questions was also rectified to avoid ambiguity and to minimize bias induced by comprehension difficulties. The speed and fluency of synchronized reading of the texts in situations 4A and 4B were modified to ensure better intelligibility. The examiners reported their remarks regarding the feasibility of the application and its appreciation by the children examined.

Thus, eight exploratory versions (updates), elaborated from January 2018 to April 2018, preceded the launch of the latest version 8.2 of the TSSI application.

#### β Study

The exploratory version of the test allowed us to judge the level of comprehension of the text and the questions, some of which were reworded to improve their intelligibility. Secondly, it enabled us to verify the relevance of the statement and the acceptability of the tool before beginning the pre-validation study. Thirdly, the β study helped us set up the scoring system of the test.

#### Pre-validation or Pilot Study

The test was administered in its digital version (total score initially set at 25) in order to verify the psychometric properties on a small sample. The average score as a function of age varied between 20/25 and 23/25 (Table 2).

**TABLE 2:** Mean TSSI score as a function of age for the pre-validation study.

**Age (years)**	**7**	**8**	**9**	**10**	**11**	**12**
Mean TSSI Score	20	20.6	20	21.5	22.2	23

### Content Validity

The same experts discussed the subdomains of ToM treated by each social situation. The six subdomains suggested were attribution of intentions, epistemic ToM, affective ToM, detection of Faux Pas, simple comprehension, and control situations. The texts and the questions were examined by experts according to whether they were clearly linked to the construct it was supposed to represent and the degree to which it exclusively represented one of the six theoretical subdomains of ToM.

The EFA also identified six subdomains that explained 69.37% of the total variance (Table 3).

**TABLE 3:** Subdomains of TSSI determined by the exploratory factorial analysis and their respective Cronbach’s alpha.

**Factors**	**1**	**2**	**3**	**4**	**5**	**6**
Subdomains Determined by factor analysis	***SCl1***	***SCl2***	***Int At***	***AToM***	***SCO***	***Ep ToM (1^*st*^ and 2^*nd*^ orders)***
Corresponding items	Item 2–0 Item 2–1 Item 2–2 Item 2–3 Item 4A–0	Item 9–0 Item 9–1 Item 9–2 Item 9–3	Item 3–0 Item 3–2	Item 6–1 Item 7–2 Item 10–0	Item 1–0 Item 5–0 Item 4A–1	Item 4A–2 Item 4B–0 Item 4B–1
Cronbach’s alpha	0.928	0.935	0.381	0.876	0.442	0.398

*SCl, social clumsiness; SCl1, social clumsiness type 1; SCl2, social clumsiness type 2; Int At, intention attribution; SCO, simple comprehension; AToM, affective ToM; Cont Sit, sontrol situation; EpToM, epistemic ToM.*

Calculated Cronbach’s alpha of each subdomain, as is shown in Table 3, revealed that our test assesses pertinently the detection of social clumsiness and attribution of affective mental states. On the contrary, attribution of intentions, epistemic ToM, and simple comprehension had low content validity.

### Construct Validity

#### Exploratory Factor Analysis

Exploratory factor analysis was performed to assess the dimensionality of the TSSI. Conducted after eliminating items with almost zero variance (variance < 0.05) (item 6–0, item 3–1, item 7–1) and those not correlated with the overall score (item 7–0, item 8–0), the EFA with reference to eigenvalues greater than 1 extracted six subdomains that explained 69.37% of the total variance with a globally similar distribution of items (Tables 3, 4).

**TABLE 4:** Pearson correlations between the subdomains of the TSSI and between each subdomain and the overall score.

**Correlations**	**SCl1**	**SCl2**	**Int At**	**AToM**	**SCO**	**EpToM**
SCl1	1	0.263**	0.052	0.187*	−0.112	0.265**
SCl2	0.263**	1	0.212*	0.230*	0.200*	0.299**
Int At	0.052	0.212*	1	0.191*	0.301**	0.221*
AToM	0.187*	0.230*	0.191*	1	0.158	0.271**
SCO	−0.112	0.200*	0.301**	0.158	1	0.091
EpToM	0.265**	0.299**	0.221*	0.271**	0.091	1
Total TSSI Score	0.556	0.666	0.397	0.52	0.301	0.704

**The correlation is significant at the 0.01 level (two-way). *The correlation is significant at the 0.05 level (two-way). SCl, social clumsiness; SCl1, social clumsiness type 1; SCl2, social clumsiness type 2; Int At, intention attribution; SCO, simple comprehension; AToM, affective ToM; EpToM, epistemic ToM.

#### Matrix of Inter-Correlation of Subdomains With the Overall Score

We found a significant correlation between all subdomains and the overall score with an r of Pearson ranging from 0.301 for factor 5 to 0.704 for factor 6 (see Table 4).

Factors 1 and 2 were significantly correlated (*p* = 0.03) with an r of Pearson of 0.263. They are two different types of the same ToM domain (social clumsiness).

Factors 1 and 2 (social clumsiness) were both correlated with factor 4 (affective ToM).

### Fidelity

#### Internal Coherence

In order to measure the degree of internal coherence of the TSSI, we used two complementary statistical methods. First, item-total correlation matrix (Table 5) showed that items 7–0 and 8–0 were not significantly correlated with the overall score and were therefore eliminated from the test. The remaining items were highly correlated with the total score.

**TABLE 5:** Item-total correlation indices.

**r item- total score**	**< 0.2**	**[0.2, 0.5]**	**≥ 0.5**
Items	Item 7–0 Item 8–0	Item 1–0 Item 2–3 Item 3–0 Item 3–2 Item 4A–0 Item 5–0 Item 7–2 Item 9–0 Item 10–0	Item 2–0 Item 2–1 Item 2–2 Item 4B–1 Item 6–1 Item 9–1 Item 9–2 Item 9–3

Second, the Cronbach’s alpha index of the total scale, after eliminating items that were uncorrelated with the overall score (items 7–0 and 8–0) and those with a variance of < 0.05 (items 6–0, 7–1, 3–1), was calculated to be 0.809.

Thus, the rectified version of the TSSI was made up of nine situations, i.e., 20 questions evaluating the detection of social clumsiness (nine items), the attribution of affective mental states (three items), epistemic ToM (three items), intention attribution (2 items) and simple comprehension (three items).

### Factors Correlated With Better Performance in TSSI

#### Age

The average TSSI score improves with age, ranging from 15.9 at age 7 to 18.91 at age 12. We also found a significant difference between the different age groups (_F_117^5_ = 3.69, *p* = 0.004). A significant improvement in the average scores of the TSSI was objectified from the age of 9 years. The evolution of scores is thereafter slower.

#### Gender

**FIGURE 1:**
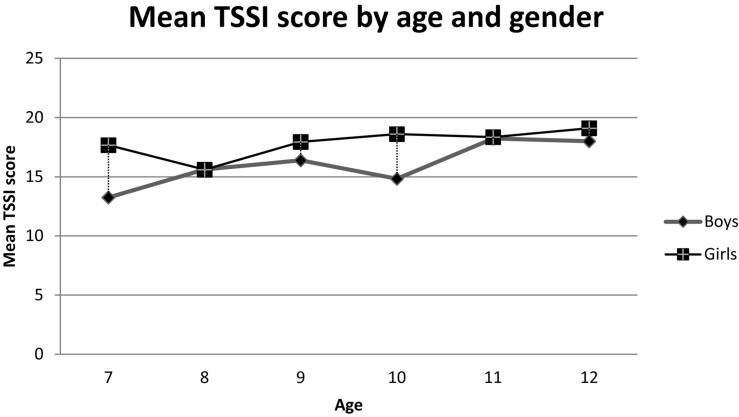
Mean TSSI score by age and gender.

Girls did better at TSSI. The mean total score in girls was 18.13 ± 2.136 compared to 16.43 ± 3.27 in boys. This difference was significant (*p* = 0.01) and was observed in all age groups (Figure 1).

#### Verbal Age

The total TSSI score was significantly correlated with verbal age (*p* ≤ 10^–3^). This correlation was positive (Rho of Spearman = 0.420). We performed a multiple linear regression to model the linear relationship between the TSSI overall score and the chronological, mental, and verbal ages of participants (see Table 6). Ruling out the role of the age, the linear model revealed a positive and significant effect of the verbal age on the overall score (*p* = 0.001, *R*^2^ = 0.278).

**TABLE 6:** Multiple linear regression of the TSSI overall score as a function of chronological, mental, and verbal ages.

**Model**	***B***	**Standard error**	**Beta**	***t***	**Sig**
Constant	4.694	1.979		2.372	**0.019**
Age	0.283	0.178	0.151	1.586	0.115
Mental age	0.339	0.154	0.185	2.194	**0.03**
Verbal age	0.621	0.187	0.330	3.319	**0.001**

*R = 0.527. *R*-squared = 0.278. Adjusted *R*-squared = 0.259. Standard error of estimation = 2.362. Results in bold are those with significant *p* value.*

#### School Performance

Children’s performance on the TSSI was not correlated with their academic year averages (*p* = 0.36).

## Discussion

The main purpose of our work is to study the psychometric characteristics of the TSSI by evaluating its validity and reliability.

### Population of Study

Our study recruited a pediatric population-based sample initially composed of 150 Tunisian children, aged 7 to 12 years, enrolled in regular schools in five states. The sex ratio (M/F) of the 123 included children was 0.8.

The size of our sample and its target age are comparable to studies carried out in the same field. For instance, the validation of the French version of the Faux Pas Test, which consists of 10 situations each containing 4 to 5 items, was based on a control group of 127 children and a group of 11 children with ASD (Garrigues, 2013). We can also cite the example of the validation study of the Picture Stories tool of Leslie and Frith (1986) and Faísca et al. (2016). Its sample consisted of 27 typically developing children (sex ratio = 1), 21 children with ASD (including 14 boys), and 15 children with Down syndrome (sex ratio = 1).

### Internal Validity of the TSSI

#### Validity of Appearance

As part of our validation process, we initially presented the exploratory version of the test, consisting of 25 questions, to a group of 7 experts and then conducted a beta test in 15 typically developing children and a pre-validation study in 20 children to verify the psychometric properties of the tool. These steps allowed us to modify the content of the test in terms of both the formulation of the scenarios and the evaluation questions and the pictures illustrating the stories and to suggest a scoring system. Similarly, the stories included in the original version of the Faux Pas Test of Baron-Cohen et al. (1999) were gathered by asking people to give examples of Faux Pas incidents from their experience. These stories were then presented to a panel of four judges (Baron-Cohen et al., 1999).

Then, we have theoretically grouped the different items according to the type of ToM being tested into six subdomains: control questions, items assessing affective ToM, items assessing epistemic ToM, items assessing the attribution of intentions, items assessing simple comprehension, and items assessing the understanding of social clumsiness.

#### Validity of Content

According to Fermanian (2005), an item is relevant if it corresponds well to the domain it is supposed to explore (Fermanian, 1996). A domain is correctly represented if it is explored by a number of items corresponding to its importance for the phenomenon studied (Anastasi, 1986).

To verify this condition, an exploratory factorial analysis with Varimax rotation was performed after eliminating items with zero or near-zero variance and those that were not correlated with the overall score. This analysis also identified six subdomains that explained 69.37% of the total variance (Table 3). Nevertheless, the distribution of the remaining items into subdomains was slightly different from their theoretical distribution into ToM subdomains (see Appendix 1).

The differences concerned five items. The first question of the 4^*th*^ situation A (item 4A-0), which reproduces the principle of Sally and Anne’s test, belongs more to the subdomain of social clumsiness than to the epistemic ToM domain. The fact that Rami hid Salma’s present in his birthday party could have been judged by the children as unsuitable, “inconvenient,” clumsy, etc.

Moreover, it turned out that situation 10, theoretically designed as a control situation, conveys emotional states of joy and enthusiasm expressed in the utterance of the protagonists of this situation (Rami’s friends admire his excellent sport performance). Its corresponding items could be thus logically classed in affective ToM. In addition, situation 5, which is supposed to test affective ToM, belongs rather to the subdomain of simple comprehension. Indeed, the metaphor pronounced by the protagonist would have been so limpid and in common use that it could have been understood as a simple expression and not a metaphorical figure of speech. Furthermore, the questions in situations 2 and 9 were theoretically grouped in the same ToM subdomain (Social Clumsiness). Factor analysis split them into two separate subdomains since they do not represent the same type of social clumsiness. Indeed, situation 2 presents the case of divulging of a secret while situation 9 deals with detecting another type of Faux Pas committed by the main character consisting in offending someone unintentionally.

The next step to verify the validity of content was to calculate the Cronbach’s alpha for each subdomain. Social clumsiness type 1, social clumsiness type 2, and affective ToM subdomains had Cronbach’s alpha greater than 0.8, while those of intention attribution, simple comprehension, and epistemic ToM had respective coefficients of 0.381, 0.442, and 0.389.

Thus, it appears that our test evaluates pertinently social clumsiness (types 1 and 2) and affective ToM. To obtain the finalized version of the TSSI, it will be just necessary to rectify the algorithm of the application by removing the items of the factors with low Crohnbach’s alpha and rectifying the global score to 12 instead of 20.

#### Validity of Construct

The evaluation of the internal structure of a test is based not only on the factor analyses but also on the correlation matrix between the subdomains and the overall score (Bolarinwa, 2015). Data analysis showed that three items had low variability and were poorly represented in the factor analysis. The items 6–0, 7–1, and 3–1, which are simple comprehension questions, were answered correctly by almost all children. Due to their low discriminatory values, they have been removed from the final version of the test.

We found a significant correlation between the factors and the overall score with an r of Pearson ranging from 0.301 for factor 5 to 0.704 for factor 6 (see Appendix 1). These results reflect the total congruence of the items.

Regarding correlations between subdomains, factors 1 and 2 were significantly correlated (*p* = 0.03) with r of Pearson of 0.263. This correlation seems to be relevant because these two factors constitute two different types of the same ToM domain (social clumsiness). These two factors were also significantly correlated with the affective ToM (factor 4) and the epistemic ToM (factor 6) subdomains, as well as with the attribution of intention (factor 3).

Indeed, in order to react in an adapted way in a given social situation, a good contextual analysis is necessary by integrating several operational schemes to correctly infer the interlocutor’s affective and/or epistemic mental states (Chabot et al., 2015). The decoding of mental states refers to the perception and identification of social cues in daily interactions (De Rosnay et al., 2014; Kanske et al., 2015a). These different elements can be, for example, a gesture, the direction of the interlocutor gaze, the prosody of his/her speech, his/her facial expressions, etc. Thus, decoding an irony (situation 6) results from the detection and confrontation of prosodic clues and emotional or behavioral expressions of the speaker in order to identify the meaning he intended (Pleah, 2016). Hence, the correlations found between these factors can be explained. The decoding mechanism associated with ToM thus covers processes of detection, integration, and comparison of clues from multimodal sources and makes it possible to define the nature of the mental state (Conte et al., 2019).

Reasoning is the second process of ToM. It enables us to understand, explain, or predict actions and requires access to information about the protagonist and the context of the social situation. Success in Faux pas detection task (Factors 1 and 2) therefore calls upon the different subdomains of Tom: Factor 3 (intention attribution), Factor 4 (affective ToM), and Factor 6 (epistemic ToM).

#### Internal Consistency of the TSSI

For our validation study, we assessed the correlation between the score of each item and the overall test score. 7–0 and 8–0 items had no significant correlation with the overall score. The corresponding questions were therefore eliminated from the test. The remaining items were highly correlated with the total score. Thus, at the end of the factor analysis and the calculation of the overall item-score correlation, the theoretical subdomain “control situations,” which grouped the respective questions for situations 1, 8, and 10, was eliminated from the test.

Situations 1, 8, and 10 were initially conceived as control situations. Statistical analysis changed this distribution. The first control situation (situation 1) was classified by the factor analysis, in the subdomain “Simple comprehension questions”; situation 8 was eliminated because its items did not have a significant correlation with the overall score. Situation 10, when we reconsider it, conveys emotional states of joy and enthusiasm expressed by the utterances of the protagonists of the situation (Rami’s friends who admire Rami’s excellent sports performance). Its inclusion in factor 4 by factorial analysis can be explained in that way.

Randomness in the categorization of control situations is not specific to our test. In literature, the control situations of the False Steps by Baron-Cohen et al. (1999) have already been criticized, especially in its adult version. Originally, the added value of control situations is to better assess a patient’s skills to discriminate between both kinds of situations (with or without Faux Pas).

Some authors consider that the inability to correctly reject non “Faux Pas” situations may indicate an impairment in ToM. Therefore, the original version of Faux Pas (Baron-Cohen et al., 1999) and its reduced version developed by Fernandes et al. (2018) and Fernández-Modamio et al. (2018) included control situations in their respective tests.

Nevertheless, when validated in Swedish and in Portuguese (Söderstrand and Almkvist, 2012; Faísca et al., 2016), control situations of the same test showed measures of very low reliability, marked ceiling effects, and a clearly asymmetrical distribution of its items in the subdomains determined by the factor analysis.

This could be explained by the fact that a better perception of social clumsiness in normal adults could lead to an over-interpretation of obvious situations (Fernandes et al., 2018). The same validation work underlines that control situation scores are unreliable as clinical indicators of good ToM skills. In fact, patients with schizophrenia usually have the same performance as control subjects in the understanding of control stories, but a clear deficit in the understanding of the Faux pas (Booules-Katri et al., 2019). In addition, *post hoc* Newman–Keuls tests revealed this was due to the group with Asperger syndrome/high-functioning autism performing significantly lower on the Faux Pas than on the Control Stories, relative to the normal group. As can be seen, the two groups did not differ on the Control Stories, both being at ceiling (Baron-Cohen et al., 1999).

We also calculated the Cronbach of the TSSI in order to judge its internal consistency. Cronbach’s alpha is a statistical index used to determine the consistency of the set of items making up a psychological test (Cronbach and Shavelson, 2004). Cronbach’s alpha index of the total scale, after eliminating items that were uncorrelated with the overall score (items 7–0 and 8–0) and those with zero or near zero variance (items 6–0, 7–1, 3–1), was calculated to be 0.809. This is in favor of a good internal consistency of our test as the commonly accepted thresholds for good reliability of a measurement tool ranges from 0.7 to 0.95 (Roberts and Priest, 2006).

Notwithstanding the relevance of our results, some limitations of this study warrant mention. Chief among them was the absence of study of sensitivity. In fact, the lack of equivalent tool validated in the Tunisian general pediatric population makes it impossible to study the sensitivity and the specificities of the TSSI.

In addition, we faced some limitations during this validation study that hampered the study of temporal stability. It was not verified since the overall procedure lasted too long, preventing the study of reproducibility of the measurements by a test–retest.

Still another limitation is that we did not include other socioeconomic factors such as educational and income levels of the parents of the participants.

The outcomes of our study deserve to be confirmed by a broader research to better trace the developmental progression of ToM and the evolution of the mean TSSI score in standard deviations. We envisage not only to enlarge the sample size but also to assess a clinical population including children with ASD. In fact, to fully understand a test’s strengths as well as its shortcomings, it is relevant to study its sensitivity.

### Factors Correlated With Better Performance in Theory of Mind

#### Age

Our work, in consistency with the data in the literature, highlights the developmental aspect of ToM acquisition. This developmental continuum is essential, as the numerous regular works in the literature on the subject underline (Bedford et al., 2012).

Frith and Frith (1999) proposed different stages of ToM acquisition (Ding et al., 2015):

Before 1 year of age, the baby shares his or her attention with that of another. Joint attention is made possible by determining the direction of the other person’s gaze. Around 18 months, the simulation situation (“pretending”) is understood. Between the ages of 2 and 4 years, a child may engage in behavior designed to deceive the other person, such as lying (Frith, 1994). At age 4, a child may picture a mistaken belief of the person he is talking to. According to Derouesné (2003) and Duval et al. (2011), the tasks of epistemic ToM are also successfully performed by a child of about 4 years of age. Otherwise, second-level epistemic ToM tests exploring belief about belief are not passed until a little later, i.e., around 6–7 years old. Ultimately, the so-called Faux Pas tests proposing to a subject to detect social clumsiness are only passed at the age of 9–11 years (Giovagnoli, 2019). This could explain the “ceiling” effect often reached in classical ToM tasks (Fischer et al., 2017) and also observed in our study.

#### Gender

Girls performed significantly better on the TSSI test: the mean overall score for girls was 18.13 ± 2.136 compared to 16.43 ± 3.27 for boys (*p* = 0.01).

The superiority and precocity of girls’ performances in the theory of mind could be explained by some cultural aspects of our population of study. Indeed, the transgenerational speech of grandmothers addressed to their granddaughters, especially in Tunisian extended families, contains recipes of feminine social skills: how to be sensitive to the benevolent or malevolent intentions of others in order to be able to protect herself and how to decode the emotions and thoughts of other family members in order to anticipate their reaction, in an attempt to forge them to fulfill their social roles as wife and mother. Girls are generally more to this “oral social learning” and passionate about these stories. They are therefore more initiated to the detection of social clumsiness.

Moreover, girls are more involved in their mothers’ conversation circles with friends and neighbors. They learn how to exchange flattery, how to brighten up the conversation with anecdotes and jokes, to stoke the atmosphere with irony or sarcasm. Tunisian girls are also more passionate about films and soap operas. These film productions illustrate everyday social scenes and could stimulate social intelligence.

Recently, Taumoepeau et al. (2019) conducted a study of sixty Iranian and New Zealand mermaids and their children, examining cross-cultural differences in the effect of parent–child conversations. Mothers completed a task of describing a picture book without words; children were asked to complete a battery of five tasks related to the theory of mind. The difference in performance between Iranian and New Zealand children can be explained by the different strategies used. Iranian mothers referred more to other people’s desires and mental states, while New Zealand mothers referred more to child-directed cognitions and mental states. Children who performed better on various belief tasks had mothers who referred more to their own mental states (Taumoepeau et al., 2019).

In the literature, gender differences are often reported, particularly in the area of affective ToM (Fischer et al., 2017). Girls acquire and master ToM concepts earlier than boys (Larzul, 2010).

In the validation of the original English version False Steps of Baron-Cohen et al. (1999), an association between overall score and gender was reported, with a significant difference between the mean score of girls and boys. In the validation study of the French version of Baron–Cohen’s false steps, which was passed to 127 neurotypical children aged 7 to 11 years, a better average score was found for girls, though not reaching significance (Garrigues, 2013). This difference in social background and social experience may “boost” girls to acquire ToM earlier than boys.

Baron-Cohen (2002) postulated that the male brain is psychometrically defined to perform better in systematization than in empathy, unlike the female brain, which is characterized by a different cognitive profile (Lawson et al., 2004). Empathy, in its cognitive component, can be layered on affective ToM, allowing the mental representation of other people’s emotions or feelings and sharing them (Kanske et al., 2015b). Systematizing means analyzing the components of a system and deducing the rules that govern its functioning in order to control it. Systematization is a powerful way to understand the functioning of an “inanimate” universe, whereas empathy is an essential social skill for understanding and predicting social interactions. These hypotheses could explain the superiority of female performance in attributing mental states to others and predict their behavior.

In adults, neuroimaging research suggests that, compared to men of the same age, women use additional brain regions that underlie emotions and self-referential thinking during ToM tasks (Apperly et al., 2009).

#### Verbal Age

The overall TSSI score was positively correlated with verbal age (*p* = 0.004; Spearman’s Rho = 0.420), which is to be expected since both skills improve with age (Giovagnoli, 2019). This suggests that all children were able to cope with the syntactic requirements of the stories. Thus, failure on the TSSI tasks can hardly be attributed to poor language skills in itself. Comparatively, a significant correlation was also found between verbal age and performance on the Baron–Cohen Faux Pas Test in its French version (Garrigues, 2013).

This correlation can be explained by the progressive and relatively interdependent maturation of superior functions (language and reasoning). Given the level of complexity of ToM and the strong involvement of language in its tasks, the relationship between these two cognitive systems has been the subject of many studies (Durrleman and Franck, 2015) which highlighted the relationship between language development and the acquisition of ToM (Duval et al., 2011). Samson and Humphreys (Kanske et al., 2015b) point out that children’s performance on false belief tasks is associated with the development of language semantics and that a delay in its acquisition may be associated with a delay in the development of ToM. Although there is conflicting data, it is accepted that language and ToM have a two-way interactive relationship.

#### School Performance

The lack of correlation between school achievement and ToM performance is not consistent with the literature on the subject. Theoretically, mindreading may be important for success in school because it affects children’s social relationships and behaviors which, in turn, are closely linked to academic achievement (Lecce et al., 2017). In fact, children’s social understanding could sustain them in building positive relationships within the class by reducing the risk of peer rejection and antisocial behavior, and this social competence, in turn, helps children to capitalize on learning situations and to increase school achievement. Generally speaking, children’s abilities to cope with the social environment in the early school years are important factors in predicting academic success. Indeed, children at school are exposed to a new community of unfamiliar peers and adults, and the extent to which they are equipped with the socio-cognitive abilities necessary to fit into this new social environment is crucial for their school success.

The lack of correlation between school achievement and performance in ToM in our study could be explained by the fact that the Tunisian education system is focused on academic rather than social acquisition. The assessment of academic achievement is mainly based on an evaluation of scientific academic knowledge and that human sciences are not taught before reaching the college level.

## Conclusion

According to our findings, we suggest that the TSSI has good psychometric qualities. Its factor structure and the significant inter-correlation between subdomains and the global score were in favor of a good construct validity. The internal consistency study showed good reliability of the final version of the TSSI (alpha of Cronbach at 0.809). Regarding the performance of children at the TSSI, we have noticed a significant association between the global score, age, and verbal intelligence. The theoretical consequences of the study of validation of the TSSI consisted in obtaining a finalized version assessing pertinently social clumsiness detection and affective ToM. Apart from the main limitation related to the lack of test–retest assessment, this work offers valuable insights about ToM and provides clinicians with a reliable tool to assess these skills in typically developing children.

## Data Availability Statement

The raw data supporting the conclusions of this article will be made available by the authors, without undue reservation.

## Ethics Statement

The studies involving human participants were reviewed and approved by Ethic committee of Razi Hospital. Written informed consent to participate in this study was provided by the participants’ legal guardian/next of kin.

## Author Contributions

OR: elaboration of the test and its administration, statistical analysis, and redaction of the article. SH: elaboration of the test, statistical analysis, and correction of the article. MG, AT, and MM: administration of the test. ZA and MH: elaboration of the test. HY and MT: choice of the pre-test. RF: elaboration of the research protocol, statistical analysis, and correction of the article. AB: elaboration of the test and the research protocol and correction of the article. All authors contributed to the article and approved the submitted version.

## Conflict of Interest

The authors declare that the research was conducted in the absence of any commercial or financial relationships that could be construed as a potential conflict of interest.

## Abbreviations

ASD: autism spectrum disorder
AToM: affective ToM
Cont Sit: control situation
EDEI-R: Differential Scale of Intellectual Efficiency
EFA: exploratory factor analysis
EpToM: epistemic ToM
F, F: female
Int At: intention attribution
M: male
MANOVA: multivariate analysis of variance
Mini-Kiddie-SADS: Mini International Neuropsychiatric Interview for Children and Adolescents-Schedule for Affective Disorders and Schizophrenia for School aged Children
p: probability value
r: Pearson s correlation coefficient
SC: social cognition
SCl: social clumsiness
SCl1: social clumsiness type 1
SCl2: social clumsiness type 2
SCO: simple comprehension
SPSS: Statistical Package for Social Sciences
ToM: theory of mind
TSSI: Tunisian Social Situation Instrument.

## References

[B1] Anastasi A. (1986). Evolving concepts of test validation. *Annu. Rev. Psychol.* 37 1–16. 10.1146/annurev.ps.37.020186.000245

[B2] Apperly I. A. Samson D. Humphreys G. W. (2009). Studies of adults can inform accounts of theory of mind development. *Dev. Psychol.* 45 190–198. 10.1037/a0014098 19210001

[B3] Baron-Cohen S. O’Riordan M. Jones R. Stone V. Plaisted K. (1999). A new test of social sensitivity: detection of faux pas in normal children and children with Asperger syndrome. *J. Autism Dev. Disord.* 29 407–418. 10.1023/A:1023035012436 10587887

[B4] Baron-Cohen S. (2002). The extreme male brain theory of autism. *Trends Cogn. Sci.* 6 248–254. 10.1016/S1364-6613(02)01904-6 12039606

[B5] Beaudoin C. Leblanc É Gagner C. Beauchamp M. H. (2020). Systematic review and inventory of theory of mind measures for young children. *Front. Psychol.* 10:2905. 10.3389/fpsyg.2019.02905 32010013 PMC6974541

[B6] Bedford R. Elsabbagh M. Gliga T. Pickles A. Senju A. Charman T. (2012). Precursors to social and communication difficulties in infants at-risk for autism: gaze following and attentional engagement. *J. Autism Dev. Disord.* 42 2208–2218. 10.1007/s10803-012-1450-y 22278030

[B7] Ben Rejeb R. (2003). *Les Echelles Différentielles d’Efficiences Intellectuelles, forme arabe (EDEI-A).* Tunis: Cogerh Sélection.

[B8] Benson J. B. Haith M. M. (2010). *Social and Emotional Development in Infancy and Early Childhood.* Denver: Academic Press.

[B9] Białecka-Pikul M. Kołodziejczyk A. Bosacki S. (2017). Advanced theory of mind in adolescence: do age, gender and friendship style play a role? *J. Adolesc.* 56 145–156. 10.1016/j.adolescence.2017.02.009 28237631

[B10] Bolarinwa O. A. (2015). Principles and methods of validity and reliability testing of questionnaires used in social and health science researches. *Niger. Postgrad. Med. J.* 22 195–197. 10.4103/1117-1936.173959 26776330

[B11] Booules-Katri T. M. Pedreño C. Navarro J. B. Pamias M. Obiols J. E. (2019). Theory of Mind (ToM) performance in high functioning Autism (HFA) and schizotypal-schizoid personality disorders (SSPD) Patients. *J. Autism Dev. Disord.* 49 3376–3386. 10.1007/s10803-019-04058-1 31104261

[B12] Bosco F. M. Gabbatore I. Tirassa M. Testa S. (2016). Psychometric properties of the theory of mind assessment scale in a sample of adolescents and adults. *Front. Psychol.* 7:566. 10.3389/fpsyg.2019.00566 27242563 PMC4860419

[B13] Brothers L. Ring B. (1992). A neuroethological framework for the representation of minds. *J. Cogn. Neurosci.* 4 107–118. 10.1162/jocn.1992.4.2.107 23967887

[B14] Chabot A. Achim J. Terradas M. M. (2015). La capacité de mentalisation de l’enfant à travers le jeu et les histoires d’attachement à compléter: perspectives théorique et clinique. *Psychiatr. Enfant.* 58 207–240. 10.3917/psye.581.0207 18052372

[B15] Conte E. Ornaghi V. Grazzani I. Pepe A. Cavioni V. (2019). Emotion knowledge, theory of mind, and language in young children: testing a comprehensive conceptual model. *Front. Psychol.* 10:2144. 10.3389/fpsyg.2019.02144 31607984 PMC6761293

[B16] Coricelli G. (2005). Two-levels of mental states attribution: from automaticity to voluntariness. *Neuropsychologia* 43 294–300. 10.1016/j.neuropsychologia.2004.11.015 15707913

[B17] Cronbach L. J. Shavelson R. J. (2004). My current thoughts on coefficient alpha and successor procedures. *Educ. Psychol. Measur.* 64 391–418. 10.1177/0013164404266386

[B18] De Rosnay M. Fink E. Begeer S. Slaughter V. Peterson C. (2014). Talking theory of mind talk: young school-aged children’s everyday conversation and understanding of mind and emotion. *J. Child Lang.* 41 1179–1193. 10.1017/S0305000913000433 24229511

[B19] Derouesné C. (2003). Théorie de l ’esprit, empathie et… bâillement. *Psychol. Neuropsychiatr. Viei.* 1 285–287. 15688536

[B20] Ding X. P. Wellman H. M. Wang Y. Fu G. Lee K. (2015). Theory-of-mind training causes honest young children to lie. *Psychol. Sci.* 26 1812–1821. 10.1177/0956797615604628 26431737 PMC4636928

[B21] Durrleman S. Franck J. (2015). Exploring links between language and cognition in autism spectrum disorders: complement sentences, false belief, and executive functioning. *J. Commun. Disord.* 54 15–31. 10.1016/j.jcomdis.2014.12.001 25637130

[B22] Duval C. Piolino P. Bejanin A. Laisney M. Eustache F. Desgranges B. (2011). La théorie de l’esprit : aspects conceptuels, évaluation et effets de l’âge. *Rev. Neuropsychol.* 3 41–51. 10.3917/rne.031.0041 18052372

[B23] Faísca L. Afonseca S. Brüne M. Gonçalves G. Gomes A. Martins A. T. (2016). Portuguese adaptation of a faux pas test and a theory of mind picture stories task. *Psychopathology* 49 143–152. 10.1159/000444689 27271151

[B24] Farrar M. J. Maag L. (2002). Early language development and the emergence of a theory of mind. *First Lang.* 22 197–213. 10.1177/014272370202206504

[B25] Fermanian J. (1996). Evaluating correctly the validity of a rating scale: the numerous pitfalls to avoid. *Rev. Epidemiol. Sante Publique* 44 278–286. 8766986

[B26] Fermanian J. (2005). Validation of assessment scales in physical medicine and rehabilitation: how are psychometric properties determined? *Ann. Readapt. Med. Phys.* 48 281–287. 10.1016/j.annrmp.2005.04.004. 15923054

[B27] Fernandes J. M. Cajão R. Lopes R. Jerónimo R. Barahona-Corrêa J. B. (2018). Social cognition in schizophrenia and autism spectrum disorders: a systematic review and meta-analysis of direct comparisons. *Front. Psychiatry* 9:504. 10.3389/fpsyg.2019.00504 30459645 PMC6232921

[B28] Fernández-Modamio M. Arrieta-Rodríguez M. Bengochea-Seco R. Santacoloma-Cabero I. Gómez de Tojeiro-Roce J. García-Polavieja B. (2018). Faux-Pas test: a proposal of a standardized short version. *Clin. Schizophr. Relat. Psychoses* 12 10–12. 10.3371/CSRP.FEAR.061518 29944413

[B29] Fischer A. L. O’Rourke N. Loken Thornton W. (2017). Age differences in cognitive and affective theory of mind: concurrent contributions of neurocognitive performance, sex, and pulse pressure. *J. Gerontol. Series B* 72 71–81. 10.1093/geronb/gbw088 27503390

[B30] Frith U. (1994). Autism and theory of mind in everyday life. *Soc. Dev.* 3 108–124. 10.1111/j.1467-9507.1994.tb00031.x

[B59] Frith U. Frith C. D. (2003). Development and neurophysiology of mentalizing. *Philos. Trans. R. Soc. Lond. B Biol. Sci.* 358, 459–473. 10.1098/rstb.2002.1218 12689373 PMC1693139

[B31] Frith C. D. Frith U. (1999). Interacting minds–a biological basis. *Science* 286 1692–1695. 10.1126/science.286.5445.1692 10576727

[B32] Gallagher H. L. Frith C. D. (2003). Functional imaging of ‘theory of mind’. *Trends Cogn. Sci.* 7 77–83. 10.1016/S1364-6613(02)00025-6 12584026

[B33] Garrigues É (2013). *Traduction et Adaptation du Faux Pas Test et Faits Cliniques*, Ph. D thesis, Academy of Paris, Paris.

[B34] Giovagnoli A. R. (2019). Theory of mind across lifespan from ages 16 to 81?years. *Epilepsy Behav.* 100:106349. 10.1016/j.yebeh.2019.05.044 31375413

[B35] Girli A. Tekin D. (2010). Investigating false belief levels of typically developed children and children with autism. *Procedia Soc. Behav. Sci.* 2 1944–1950. 10.1016/j.sbspro.2010.03.261

[B36] Hamilton K. Hoogenhout M. Malcolm-Smith S. (2016). Neurocognitive considerations when assessing theory of mind in autism spectrum disorder. *J. Child Adolesc. Ment. Health* 28 233–241. 10.2989/17280583.2016.1268141 27998262

[B37] Happé F. G. (1994). An advanced test of theory of mind: understanding of story characters’ thoughts and feelings by able autistic, mentally handicapped, and normal children and adults. *J. Autism Dev. Disord.* 24 129–154. 10.1007/BF02172093 8040158

[B38] Hughes C. Devine R. T. Ensor R. Koyasu M. Mizokawa A. Lecce S. (2014). Lost in translation? Comparing British, Japanese, and Italian children’s theory-of-mind performance. *Child Dev. Res.* 2014 1–10. 10.1155/2014/893492

[B39] Hutchins T. L. Prelock P. A. Morris H. Benner J. LaVigne T. Hoza B. (2016). Explicit vs. applied theory of mind competence: a comparison of typically developing males, males with ASD, and males with ADHD. *Res. Autism Spectr. Disord.* 21 94–108. 10.1016/j.rasd.2015.10.004

[B40] Jolliffe T. Baron-Cohen S. (1999). The strange stories test: a replication with high-functioning adults with autism or Asperger syndrome. *J. Autism Dev. Disord.* 29 395–406. 10.1023/A:1023082928366 10587886

[B41] Kanske P. Böckler A. Singer T. (2015a). Models, mechanisms and moderators dissociating empathy and theory of mind. *Curr. Top. Behav. Neurosci.* 30 193–206. 10.1007/7854_2015_412 26602249

[B42] Kanske P. Böckler A. Trautwein F. M. Singer T. (2015b). Dissecting the social brain: introducing the EmpaToM to reveal distinct neural networks and brain-behavior relations for empathy and Theory of Mind. *Neuroimage* 122 6–19. 10.1016/j.neuroimage.2015.07.082 26254589

[B43] Larzul S. (2010). *The Role of Theories of Mind Development in the Social Adaptation and the Success at School of Children 4 to 6 Years Old*, Ph. D thesis, Rennes 2 University, Rennes.

[B44] Lawson J. Baron-Cohen S. Wheelwright S. (2004). Empathising and systemising in adults with and without Asperger Syndrome. *J. Autism Dev. Disord.* 34 301–310. 10.1023/B:JADD.0000029552.42724.1b 15264498

[B45] Lecce S. Caputi M. Pagnin A. Banerjee R. (2017). Theory of mind and school achievement: the mediating role of social competence. *Cogn. Dev.* 44 85–97. 10.1016/j.cogdev.2017.08.010

[B46] Mahy C. Moses L. Pfeifer J. (2014). How and where: theory-of-mind in the brain. *Dev. Cogn. Neurosci.* 9 68–81. 10.1016/j.dcn.2014.01.002 24552989 PMC6989753

[B47] Mc Grath M. (2009). *Assessing the Effect of Culture On theory of Mind Performance in South AFRICAN Students.* Ph. D thesis, University of Cape Town, Rondebosch.

[B48] Pleah C. (2016). *Théorie de L’esprit et Compréhension de L’ironie chez les Enfants Entendants et Malentendants. Sciences Cognitives.* Ph. D thesis, University of Caen Normandie, Caen.

[B49] Premack D. Woodruff G. (1978). Chimpanzee problem-solving: a test for comprehension. *Science* 202 532–535. 10.1126/science.705342 705342

[B50] Roberts P. Priest H. (2006). Reliability and validity in research. *Nurs. Stand.* 20 41–45. 10.7748/ns.20.36.41.s58 16872117

[B51] Scholl B. J. Leslie A. M. (1999). Modularity, development and ‘Theory of Mind’. *Mind Lang.* 14 131–153. 10.1111/1468-0017.00106

[B52] Schurz M. Radua J. Aichhorn M. Richlan F. Perner J. (2014). Fractionating theory of mind: a meta-analysis of functional brain imaging studies. *Neurosci. Biobehav. Rev.* 42 9–34. 10.1016/j.neubiorev.2014.01.009 24486722

[B53] Söderstrand P. Almkvist O. (2012). Psychometric data on the eyes test, the faux pas test, and the dewey social stories test in a population-based swedish adult sample. *Nord. Psychol.* 64 30–43. 10.1080/19012276.2012.693729

[B54] Taumoepeau M. Sadeghi S. Nobilo A. (2019). Cross-cultural differences in children’s theory of mind in Iran and New Zealand: the role of caregiver mental state talk. *Cogn. Dev.* 51 32–45. 10.1016/j.cogdev.2019.05.004

[B55] Vaske J. J. Beaman J. Sponarski C. C. (2017). Rethinking internal consistency in Cronbach’s alpha. *Leis. Sci.* 39 163–173. 10.1080/01490400.2015.1127189

[B56] Wang Z. Devine R. T. Wong K. K. Hughes C. (2016). Theory of mind and executive function during middle childhood across cultures. *J. Exp. Child Psychol.* 149 6–22. 10.1016/j.jecp.2015.09.028 26592766

[B57] White S. Hill E. Happé F. Frith U. (2009). Revisiting the strange stories: revealing mentalizing impairments in autism. *Child Dev.* 80 1097–1117. 10.1111/j.1467-8624.2009.01319.x 19630896

[B58] Williams R. G. Klamen D. A. McGaghie W. C. (2003). Cognitive, social and environmental sources of bias in clinical performance ratings. *Teach. Learn. Med.* 15 270–292. 10.1207/S15328015TLM1504_11 14612262

