# Supplementary figures and images for: Validation of the Tunisian Social Situation Instrument in the General Pediatric Population
Source: Front Psychol. 2020 Oct 29;11:557173. doi: 10.3389/fpsyg.2020.557173 (PMC7658408; doi:10.3389/fpsyg.2020.557173)

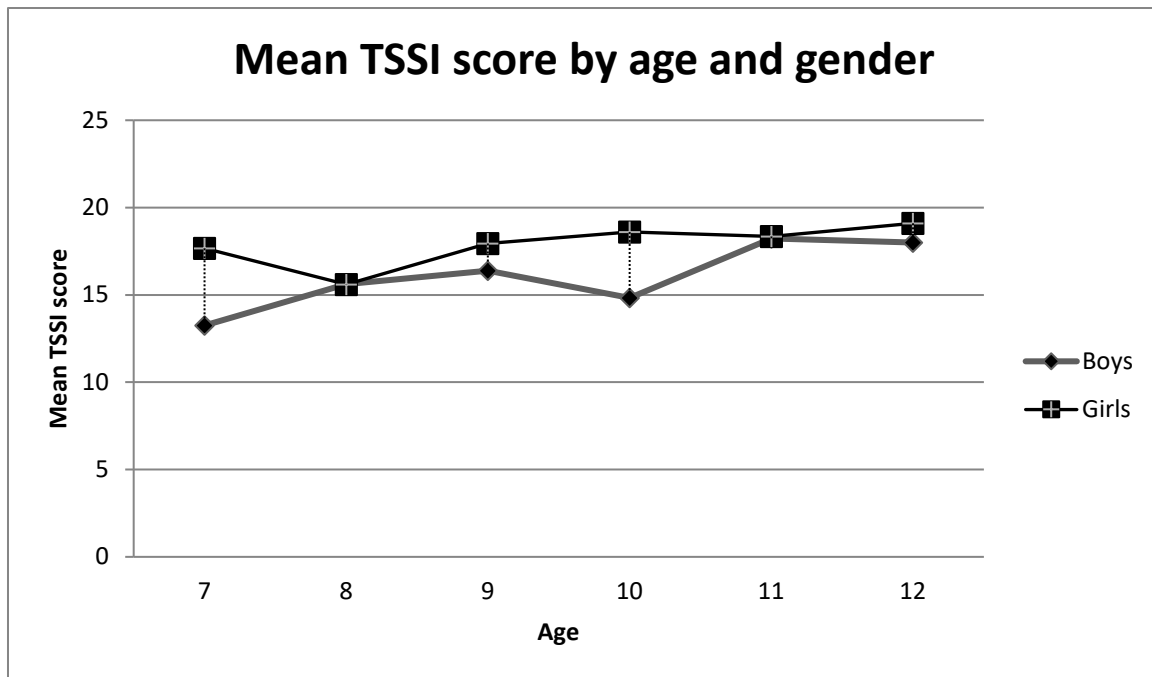

Figure N°1: Mean TSSI Score (out of 20) by age and gender (standard deviations)

Supplement: Supplementary file 1 [file Image_1.pdf]
