# Supplementary material for: Validation of the Tunisian Social Situation Instrument in the General Pediatric Population
Source: Front Psychol. 2020 Oct 29;11:557173. doi: 10.3389/fpsyg.2020.557173 (PMC7658408; doi:10.3389/fpsyg.2020.557173)
Supplement: Supplementary file 2 [file Data_Sheet_1.docx]

**Appendix 1:** Comparative table of the theoretical distribution of the items and that determined by the factor analysis

|  |  | Theoritical Distribution | Distribution by factor analysis |
| --- | --- | --- | --- |
| Situation 1/ Topic | Salma’s mother invites Rami and Myriam to Salma’s birthday party and askes them not to tell her about this surprise. |  |  |
| It 1-0 | Faux Pas detection | Cont Sit | SCO |
| Situation 2/Topic | Myriam almost reveals the birthday surprise to Salma |  |  |
| It 2-0 | Faux Pas detection | SCl | S Cl1 |
| It 2-1 | Identification of the protagonist who committed the Faux Pas | SCl | S Cl1 |
| It 2-2 | Identification of the content of the Faux Pas | SCl | S Cl1 |
| It 2-3 | Justification | SCl | SCl1 |
| Situation 3 /Topic | Rami chooses Salma’s birthday present (a teddy bear) |  |  |
| It 3-0 | Did he make the good choice? | Int At | Int At |
| It 3-1 | What did he buy for Salma? | SCO | ** |
| It 3-2 | Justification (why did he buy a teddy bear and not a ball?) | Int At | Int At |
| Situation 4-A/Topic | Rami hides Salma’s birthday present (inspired from Sally and Anne Test first order) |  |  |
| It 4A-0 | Where did Salma out the present at first? | SCO | SCl 1 |
| It 4A-1 | Where did Rami move it afterwards | SCO | SCO |
| It 4A-2 | Where will Salma look for the present when she comes back? | EpToM | EpToM |
| Situation 4-B/Topic | Salma was on the outlook for Rami when moving the birthday present (inspired from Sally and Anne test reviewed by A.Rouvière) |  |  |
| It 4B-0 | Where will Salma look for the birthday present? | EpToM | EpTOM |
| It 4B-1 | Where would Rami think Salma is going to look for the present? | EpToM | EpToM |
| Situation 5/ Topic | Stylistic Device: Metaphor |  |  |
| It 5-0 | To explain the metaphor | AToM | SCO |
| Situation 6/Topic | Salma didn’t mind her mother’s efforts in preparing the birthday cake. |  |  |
| It 6-0 | What did Salma’s mother prepare for the birthday party? | SCO | ** |
| It 6-1 | Detecting irony | A ToM | A ToM |
| Situation 7/ Topic | Salma is unsatisfied with the present of Rami but she complements him. |  |  |
| It 7-0 | Faux Pas detection | AToM | * |
| It 7-1 | Comprehension question (Is Salma satisfied with the present of Rami?) | SCO | ** |
| It 7-2 | Justification question | A ToM | A ToM |
| Situation 8/Topic | Salma is satisfied with the present of Myriam. |  |  |
| It 8-0 | Faux Pas detection | Cont Sit | * |
| Situation 9/ Topic | Salma forgot that Rami offered her the teddy bear and confessed that she tore it up because she didn’t like it. |  |  |
| It 9-0 | Faux Pas detection | SCl | SCl2 |
| It 9-1 | Identification of the protagonist who committed the Faux Pas | SCl | SCl2 |
| It 9-2 | Identification of the content of the Faux Pas | SCl | S Cl2 |
| It 9-3 | Justification question | SCl | SCl2 |
| Situation 10/Topic | Rami got upset about Salma’s attitude and went out to play football with his friends. He made a great save in the last minute of the game. |  |  |
| It 10-0 | Faux Pas detection | ContSit | AToM |

Notes: SCl: Social Clumsiness/ SCl1: Social Clumsiness type 1/SCl2: Social Clumsiness type 2

Int At: Intention Attribution

SCO: Simple comprehension

AToM : Affective ToM

Cont Sit: Control Situation

Epistemic ToM: EpToM

*: Eliminated (its correlation to the overall-score <0.2)

**: Eliminated (variance<0.05)
