# Supplementary material for: Validation of the Tunisian Social Situation Instrument in the General Pediatric Population
Source: Front Psychol. 2020 Oct 29;11:557173. doi: 10.3389/fpsyg.2020.557173 (PMC7658408; doi:10.3389/fpsyg.2020.557173)
Supplement: Supplementary file 3 [file Data_Sheet_2.docx]

| **Situations** | **Items**  **of the initial  version** | **Initial cotation** | **Items**  **of the  rectified version** | **Rectified cotation** |
| --- | --- | --- | --- | --- |
| Situation 1 | It 1-0 | 0/1 | It 1-0 | 0/1 |
| Situation 2 | It 2-0  It 2-1  It 2-2  It 2-3 | 0/1  0/1  0/1  0/1 | It 2-0  It 2-1  It 2-2  It 2-3 | 0/1  0/1  0/1  0/1 |
| Situation 3 | It 3-0  It 3-1  It 3-2 | 0/1  0/1  0/1 | It 3-0  It 3-2 | 0/1  0/1 |
| Situation 4A | It 4A-0  It 4A-1  It 4A-2 | 0/1  0/1  0/1 | It 4A-0  It 4A-1  It 4A-2 | 0/1  0/1  0/1 |
| Situation 4B | It 4B-0  It 4B-1 | 0/1  0/1 | It 4B-0  It 4B-1 | 0/1  0/1 |
| Situation 5 | It 5-0 | 0/1 | It 5-0 | 0/1 |
| Situation 6 | It 6-0  It 6-1 | 0/1  0/1 | It 6-1 | 0/1 |
| Situation 7 | It 7-0  It 7-1  It 7-2 | 0/1  0/1  0/1 | It 7-2 | 0/1 |
| Situation 8 | It 8-0 | 0/1 | It 9-0  It 9-1  It 9-2  It 9-3 | 0/1  0/1  0/1  0/1 |
| Situation 9 | It 9-0  It 9-1  It 9-2  It 9-3 | 0/1  0/1  0/1  0/1 | It 10-0 | 0/1 |
| Situation 10 | It 10-0 | 0/1 | - | - |
| Total | 10 Situations  25 items | /25 | 9 Situations  20 items | /20 |

Appendix 2: Comparaison of the items between the initial and the rectified versions of the Tunisian Social Situations Instrument
